# Influence of the external field on the excitation properties of plasmon in linear atomic chain

**DOI:** 10.1038/s41598-018-30877-w

**Published:** 2018-08-22

**Authors:** Reng-lai Wu, Jun Quan, Mengtao Sun

**Affiliations:** 1 School of Physical Science and Technology, Lingnan Normal University, Zhanjiang, 524048 P.R. China; 2 School of Mathematics and Physics, Beijing Key Laboratory for Magneto-Photoelectrical Composite and Interface Science, University of Science and Technology Beijing, Beijing, 100083 P.R. China

## Abstract

Based on the self-consistent linear response theory, the plasmon-energy absorption in linear atomic chain are studied by using the tight-binding approximation. Results indicate that the eigen-frequency of the plasmon is uninfluenced by the external electric potential, but the plasmon modes excited by various electric potentials are obviously different. Each mode of plasmon corresponds to one kind of eigen-charge distribution. When the plasmon mode is excited, the resonant charge will show a distribution characteristic the same as the one of eigen charge. And the plasmon mode can be precisely controlled by external electric potential if the eigen-charge distribution at such plasmon is known. The relationship between plasmon-energy absorption and atom number are also affected by the external electric potential. However, most of the other studies only show the normal case that the plasmon-energy absorption increases with the atom number increasing. Here, we demonstrate that the normal case commonly occurs under monotone increasing potential. And abnormal case may occur under monotone decreasing potential, ie, the plasmon-energy absorption will decrease with the atom number increasing. But, in the presence of arbitrary potential applied to the same atomic chain, the plasmon-energy absorption will always increase with the electron number increasing.

## Introduction

Study of plasmon is one of the most attractive topics in the field of optoelectronics. Now, the properties of plasmons have already been widely applied in the areas of Plasmon rulers^1^, single-molecule sensing^2^, solar energy^3^, chemical reaction^4^ and cancer therapy^5^. And plasmon will be applicated in more technical fields due to their superior properties, so the excitation properties of plasmon in nanostructures will continue to attract more attentions. In the last few decades, many researchers have investigated the excitation properties of plasmon in a lot of nanostructure systems^6–18^. They have showed that the excitation properties of plasmon are highly affected by various factors, such as the shape, size, and electron filling of nanostructures, the direction and the frequency of external field. Their results are very helpful and provide basis for the theoretical study of plasmon in nanostructure systems. In these results, plasmons are mostly observed by the dynamical electronic response of nanostructure systems, which are mostly calculated based on the electromagnetic theory^6–9^, the random-phase approximation^10–12,15,16^ and the time-dependent density functional theory^13,14,17,18^. However, the modes of plasmon calculated by these methods is not complete under the applied external electric field, for instance, the longitudinal-mode plasmon can not be excited by transverse electric field^13,14,17,18^. Thus, in our previous work, we had used eigen-equation method to investigate plasmon in atomic cluster and found a new qudarupole mode of plasmon which is not excited by uniform electric field^19,20^. Then, we have realized that the plasmon modes will be affected by external electric fields. Such property has potential application value in the field of new logic optoelectronic device because it shows opportunity for controlling the modes of plasmon by a new way, ie, changing the distribution of external electric field. Motived by this property of plasmons, this paper will further investigate the influences of external field on the excitation properties of plasmon in linear atomic chain.

Through theoretical calculations of the energy absorption spectra and the eigen function, the fundamental physical properties of plasmon in linear atomic chain are given in this paper. The results show that the eigen frequency of the plasmon is independent on the external electric potential. However, the distribution of external electric potential strongly affects the excitation of plasmon modes, since the energy that sustains the collective electronic excitation are absorbed from the external electric field. The resonance properties of plasmon are also found to be influenced by the external field, especially, the external-field has effect on the evolution of plasmon-energy absorption with the atom number. Abnormal evolution of energy absorption with atom number is discussed.

## Model and Theory

**Figure 1:**
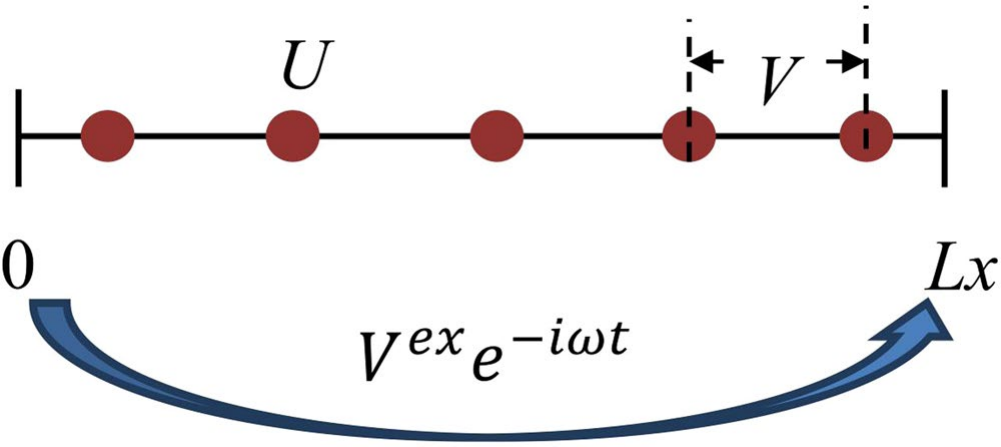
The model diagram of linear atomic chain.

The model of linear atomic chain we consider here is shown in Fig. 1, where *L*_*x*_ = (*N*_*a*_ + 1)*a* is the length of the atomic chain, *a* is the distance between the nearest two atoms, *N*_*a*_ is the atom number, *V*^*ex*^*e*^−*iωt*^ is the time-dependent external electric potential, and *V*^*ex*^ is the space-distribution part of the potential. *U* is the on-site Coulomb interaction, and only nearest-neighbor Coulomb interactions *V* is considered^21,22^.

Based on the tight-binding model and the mean field approximation, in spin-independent case, the one- band Hamiltonian of linear atomic chain system in the frequency space is

1 $$\begin{array}{ccc}{\rm{H}} & = & -{\rm{\gamma }}\sum _{l < {l}^{\text{'}}}({d}_{l}^{+}{d}_{{l}^{\text{'}}}+h.c.)+\sum _{l}{V}^{ex}(l,\omega ){d}_{l}^{+}{d}_{l}\\  &  & +\,\sum _{l}(U\delta \langle n(l,\omega )\rangle +V\sum _{{l}^{\text{'}}}\delta \langle n({l}^{\text{'}},\omega )\rangle ){n}_{l}\,\end{array}$$

where, *γ* is the nearest-neighbor transfer energy, *l* and *l′* are the positions of atoms. ${d}_{l}({d}_{l}^{+})$ is the annihilation(creation) operator on position *l*, and ${n}_{l}={d}_{l}^{+}({d}_{l})$. *δ*〈*n*(*l*, *ω*)〉 is the charge number induced by the external potential, and the total charge number is *n*(*l*, *ω*) = 〈*n*(*l*)_0_〉 + *δ*〈*n*(*l*, *ω*)〉, where 〈*n*(*l*)_0_ is the average occupied number in static case. Here, only the frequency-dependent part *δ*〈*n*(*l*, *ω*)〉 is considered because the frequency-independent part 〈*n*(*l*)〉_0_ doesn’t have effect on the dynamical response of the system. Moreover, the retardation effects arising from the distance between atoms is ignored here, since the interatomic distance is very small^23,24^.

For finite linear atomic chain, the eigen energy *E*_*n*_ and the corresponding wave function *ψ*_*n*_(*l*) can be written as

2 $${E}_{n}=\,-\,2\gamma \,\cos (\frac{n\pi }{{N}_{a}+1})\,,\,n=1,\,2,\,3\,\ldots \,{N}_{a}$$

and

3 $${\psi }_{n}(l)=\sqrt{\frac{2}{{N}_{a}+1}}\,\sin (\frac{n\pi }{{N}_{a}+1}l)$$

Based on the linear response theory, the induced charge number under perturbation *V*^*ex*^(*l*, *ω*)*e*^−*iωt*^ is

4 $$\delta \langle n(l,\omega )\rangle =\sum _{l^{\prime} }{\rm{\Pi }}(l,l^{\prime} ,\omega )[e{V}^{ex}(l^{\prime} ,\omega )+U\delta \langle n(l^{\prime} ,\,\omega )\rangle +V\sum _{l^{\prime\prime} }\delta \langle n(l^{\prime\prime} ,\,\omega )\rangle ]$$

where Lindhard function Π(*l*, *l*′, *ω*) is

5 $${\rm{\Pi }}(l,l^{\prime} ,\,\omega )=2\sum _{mn}\frac{f({E}_{m})-f({E}_{n})}{{E}_{m}-{E}_{n}-\omega -i\eta }{\psi }_{m}^{\ast }(l){\psi }_{n}(l){\psi }_{n}^{\ast }(l\text{'}){\psi }_{m}(l^{\prime} )$$

the 2 appears in the front of the equation above corresponds to two spins, *f*(*E*_*m*_) is the fermi function, parameter *η* is the scattering rate which makes the resonance of plasmon a broadening. For simplicity, Eq. (4) can be written as

6 $$\delta \langle n(l,\omega )\rangle -\sum _{l^{\prime} l^{\prime\prime} }{\rm{\Pi }}(l,l^{\prime} ,\omega ){v}_{l^{\prime} l^{\prime\prime} }\delta \langle n(l^{\prime\prime} ,\omega )\rangle =\sum _{l^{\prime} }{\rm{\Pi }}(l,l^{\prime} ,\omega )[e{V}^{ex}(l^{\prime} ,\omega )]$$

where

7 $${v}_{l^{\prime} l^{\prime\prime} }=\{\begin{array}{c}U,\,|l^{\prime} -l^{\prime\prime} |=0\\ V,\,|l^{\prime} -l^{\prime\prime} |=1\\ 0,\,\,else\end{array}$$

Take the induced charge *Q*(*l*, *ω*) = *δn*(*l*, *ω*) into the Eq. (6), have

8 $$Q(l,\omega )-\sum _{l^{\prime} l^{\prime\prime} }{\rm{\Pi }}(l,l^{\prime} ,\omega ){v}_{l^{\prime} l^{\prime\prime} }Q(l^{\prime\prime} ,\omega )={e}^{2}\sum _{l^{\prime} }{\rm{\Pi }}(l,l^{\prime} ,\omega ){V}^{ex}(l^{\prime} ,\omega )$$

Following Eq. (8), the energy absorption can be calculated by function

9 $$L({\rm{\omega }})=\frac{1}{2}\omega {\rm{Im}}\{\sum _{l}Q(l,\omega ){V}^{ex}(l,\omega )\}$$

and *L*(*ω*) shows a peak at plasmon frequency *ω*, and it should be noted that the energy absorption function depends on both the induced charge and the external potential.

Furthermore, we set *V*^*ex*^(*l*′, *ω*) = 0, and get the eigen-equation

10 $$\sum _{l^{\prime\prime} }[{\delta }_{ll^{\prime\prime} }-\sum _{l^{\prime} }{\rm{\Pi }}(l,l^{\prime} ,\omega ){v}_{l^{\prime} l^{\prime\prime} }]Q(l^{\prime\prime} ,\omega )=0$$

According to the Eq. (10), the plasmon frequency ω can be also calculated by

11 $$F({\rm{\omega }})={\rm{\det }}[{\delta }_{ll^{\prime\prime} }-\sum _{l^{\prime} }{\rm{\Pi }}(l,l^{\prime} ,\omega ){v}_{l^{\prime} l^{\prime\prime} }]=0\,$$

Here, the eigen-frequency of plasmon should be obtained by both Re(*F*(ω)) = 0 and Im(*F*(ω)) ≈ 0. If Im(*F*(ω)) ≫ 0, the collective excitation will decay rapidly into single particle excitation. So the plasmon frequency can be also found at the peak of the eigen function A(ω) = Im(1/*F*(ω)).

## Results and Discussions

In the following, most of the results are calculated in the wannier representation except the charge distribution. The distribution of charge in the real space is calculated by $Q(r,{\rm{\omega }})=\sum _{l}Q(l,\omega ){|\varphi (l,r)|}^{2}$, where *ϕ*(*l*, *r*) is a *s*-wave function which is identical to the wannier function in the tight-binding model, here, we set *ϕ*(*l*, *r*) = *R*_40_(r − *l*)*Y*_00_(*θ*, *ϕ*). In all calculations, we set parameters *U* = 3, *V* = 1, *η* = 0.01. The unit of frequency is the parameter *γ*, the unit of the space coordinate is the distance *a*. Arbitrary units are applied for the values of the eigen function, energy absorption and the charge response.

**Figure 2:**
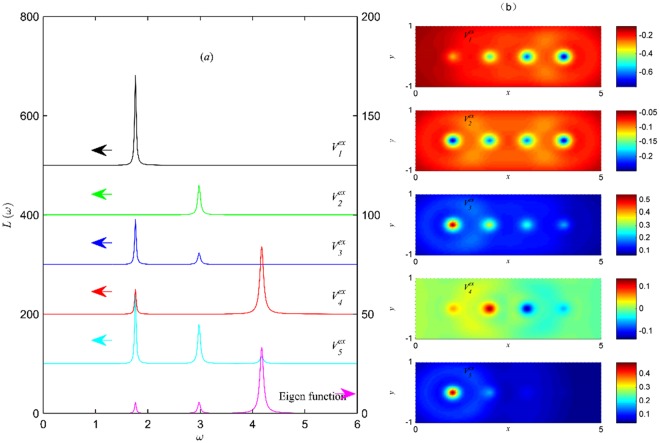
(**a**) Spectra of energy absorptions *L*(ω) (left y label) and the eigen function *A*(ω) (right y label). Where, atom number *N*_*a*_ = 4, electron number *N*_*e*_ = 2, external potentials ${V}_{1}^{ex}=\,-\,2l$, ${V}_{2}^{ex}=\,-\,\frac{2}{|l-{L}_{x}/2|}$, ${V}_{3}^{ex}=\frac{6}{l}$, ${V}_{4}^{ex}=\,-\,\frac{1}{l-{L}_{x}/2}$, ${V}_{5}^{ex}=\,-\,\frac{16}{l}{e}^{-l}$. (**b**) Distributions of potentials ${V}_{1}^{ex}-{V}_{5}^{ex}$ (top to bottom) in the real space.

First, we focus on the influence of external-field distribution on the excitation of plasmon modes, which are given by the peaks of the eigen-function and the energy absorption spectra. In Fig. 2 (right y label), the eigen-function *A*(*ω*) shows three peaks at frequencies *ω*_1_ = 1.764, *ω*_2_ = 2.982 and *ω*_3_ = 4.176, respectively, which implies three eigen-modes of plasmon in the system of *N*_*a*_ = 4, *N*_*e*_ = 2. Here, all modes of plasmon can be found by the peaks of the eigen-function, since all the plasmon frequencies are the solutions of the eigen equation^19,20,25–27^. However, the spectra of eigen function cannot be observed in experiment, so we give the spectra of energy-absorptions under potentials ${V}_{1}^{ex}-{V}_{5}^{ex}$. The external perturbations should be understood as *V*_*i*_^*ex*^*e*^*−iωt*^, *i* = 1, 2, 3, …, and all these potentials are applied in the direction along the chain. Here, the forma of all the potentials are given in the space of wannier representation. In the real space, potentials are calculated by ${V}_{i}^{ex}({\bf{r}},{\rm{\omega }})=\sum _{l}{V}_{i}^{ex}(l,\omega ){|\varphi (l,{\bf{r}})|}^{2}$, *i* = 1,2,3, …, and the distributions of potentials ${V}_{1}^{ex}-{V}_{5}^{ex}$ in the *x-y* plane are showed in Fig. 2(b). Since *s*-wave function *ϕ*(*l*, *r*) is an odd function, *V*(*r*, ω) has the same symmetry as *V*(*l*, *ω*). Different forms of potentials are chosen to excite different modes of plasmon. In the space of wannier representation, ${V}_{1}^{ex}$ is similar to a potential of uniform electric field, ${V}_{2}^{ex}-{V}_{5}^{ex}$ are potentials of non-uniform electric field, especially, ${V}_{2}^{ex}$ is a symmetrical potential, ${V}_{4}^{ex}\,\,$is an anti-symmetrical potential, ${V}_{3}^{ex}$ and ${V}_{5}^{ex}$ are asymmetrical potentials, and ${V}_{5}^{ex}$ is similar to the Yukawa potential. To display the energy-absorption peaks under different electric potentials at the same time, the amplitudes of various potentials are given with appropriate values. In Fig. 2 (left y label), the energy absorptions are changed with the frequency of external potentials, if applying potential ${V}_{1}^{ex}$ to the system, there is only one energy-absorption peak at frequency *ω*_1_, which implies that only the mode of plasmon at frequency *ω*_1_ is excited by ${V}_{1}^{ex}$. And even if the frequency of ${V}_{1}^{ex}$ equals to *ω*_2_ or *ω*_3_, the value of energy-absorptions is zero, which obviously implies the two modes of plasmon at frequencies *ω*_2_ and *ω*_3_ can not be excited by potential $\,{V}_{1}^{ex}$. Similarly, this figure also show that only the mode of plasmon at frequency *ω*_2_ is excited by ${V}_{2}^{ex}$, only two modes of plasmon at frequencies *ω*_1_ and *ω*_2_ are excited by ${V}_{3}^{ex}$, only two modes of plasmon at frequencies *ω*_1_ and *ω*_2_ are excited by ${V}_{4}^{ex}$, and all three modes of plasmon at frequencies *ω*_1_, *ω*_2_ and *ω*_3_ are excited by ${V}_{5}^{ex}$. These results can be also understood as: the mode of plasmon at frequency *ω*_1_ can not be excited by potential ${V}_{2}^{ex}$; the mode of plasmon at frequency *ω*_2_ can not be excited by potentials ${V}_{1}^{ex}$ and ${V}_{{\rm{4}}}^{ex}$; the mode of plasmon at frequency *ω*_3_ can not be excited by potentials ${V}_{1}^{ex}$, ${V}_{2}^{ex}\,$and ${V}_{3}^{ex}$. Such results show opportunity to control the plasmon modes by external electric potential. To control the plasmon mode, we must know whether the plasmon mode can be excited by an external potential or not? What kind of potential is needed to excite a plasmon mode. To answer these questions, this paper will further investigate the response of charge to the external field and show the answer by analyzing the charge distribution and the energy absorption at plasmon.

**Figure 3:**
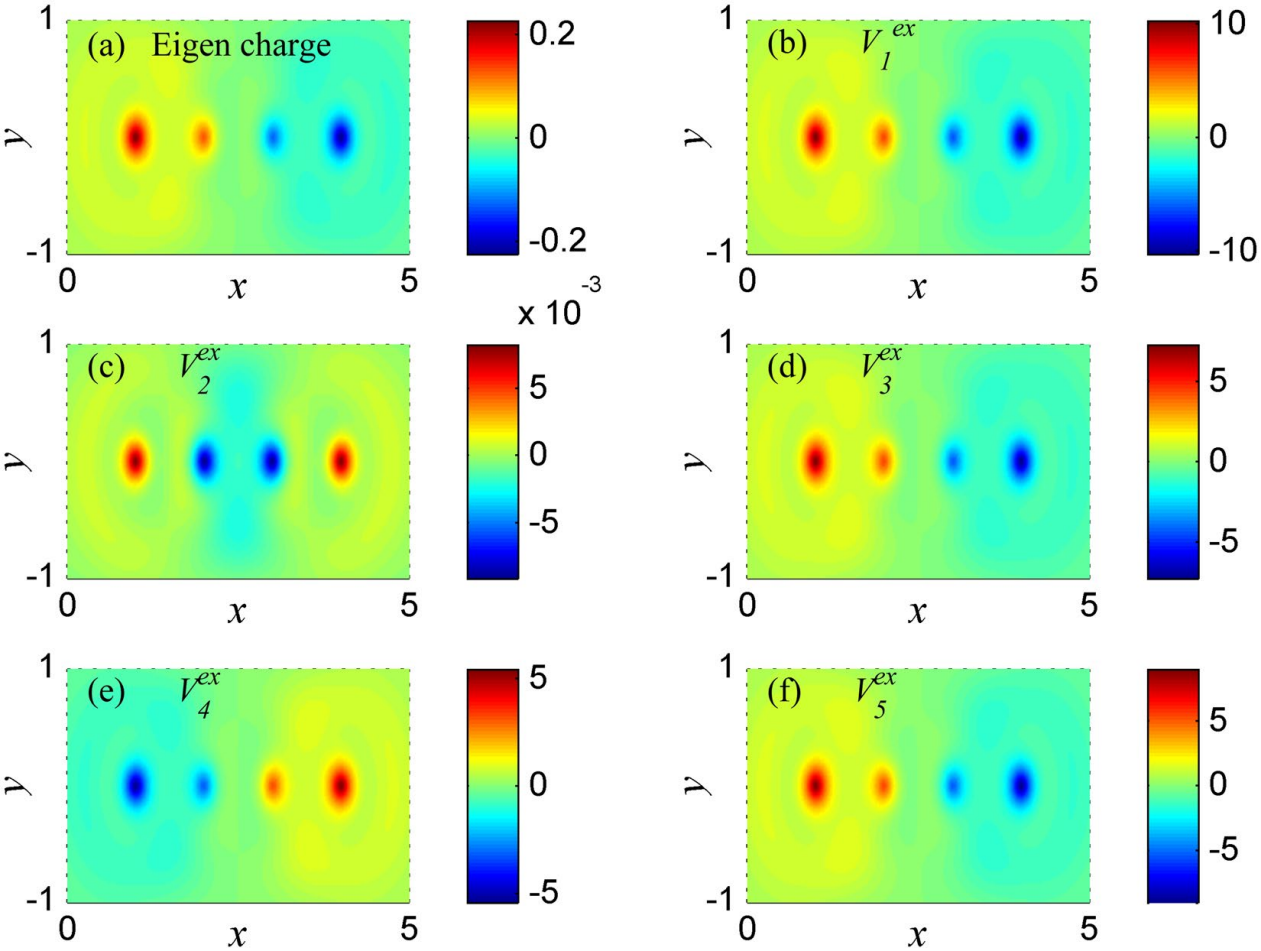
The charge distribution in the system of Fig. 2 at plasmon frequency *ω*_1_ = 1.764. (**a**) The eigen charge. (**b–f**) The charge induced respectively by external potentials ${V}_{1}^{ex}-{V}_{5}^{ex}$.

In Fig. 3(a), the eigen-charge distribution is showed at plasmon frequency *ω*_1_. The eigen charge ${Q}^{eg}(r,\omega )=\sum _{l^{\prime\prime} }{Q}^{eg}(l^{\prime\prime} ,\omega ){|\varphi (l^{\prime\prime} ,r)|}^{2}$, where *Q*^*eg*^(*l*′, *ω*) are calculated by Eq. (10) by setting *Q*(*l*″ = 1, *ω*) = 1. *Q*^*eg*^(*l*″, *ω*) is the eigenvector solution of Eq. (10), so each plasmon mode corresponding to one kind of eigen charge which is independent on the external potential. Here, only the real part of the charge is given in Fig. 3(a) (in the following figures, only the real parts of charge are plotted too, because the distribution of the imaginary parts of the charge is like the one of the real parts). In Fig. 3(a), the positive and negative charges asymmetrically gather in two sides of the atomic chain, respectively. Such eigen charge has dipole moment along the chain direction, which implies that the plasmon mode at frequency *ω*_1_ is a dipole mode. Figure 3(b–f) show the distributions of charge at frequency *ω*_1_ under potentials ${V}_{1}^{ex}$ − ${V}_{5}^{ex}$, respectively. In Fig. 3(b,d–f), the amplitudes of the charges are very large. Combine with the energy absorption peaks at *ω*_1_ in Fig. 1, we can know that the charges under potentials ${V}_{1}^{ex}$, ${V}_{3}^{ex}$, ${V}_{4}^{ex}$ and ${V}_{5}^{ex}$ are resonated at frequency *ω*_1_. And the distributions of these resonated charges are all characterized by dipole moment, which are like that of eigen charge at *ω*_1_. However, in Fig. 3(c), the charge distribution under potential ${V}_{2}^{ex}$ is symmetric among the atomic chain, which is different from the eigen-charge distribution at *ω*_1_. And the amplitude of the charge is very small, since the charge under potential $\,{V}_{2}^{ex}$ is not resonated at frequency *ω*_1_. The non-resonant charge shows symmetric distribution because it is polarized by symmetric potential $\,{V}_{2}^{ex}$.

**Figure 4:**
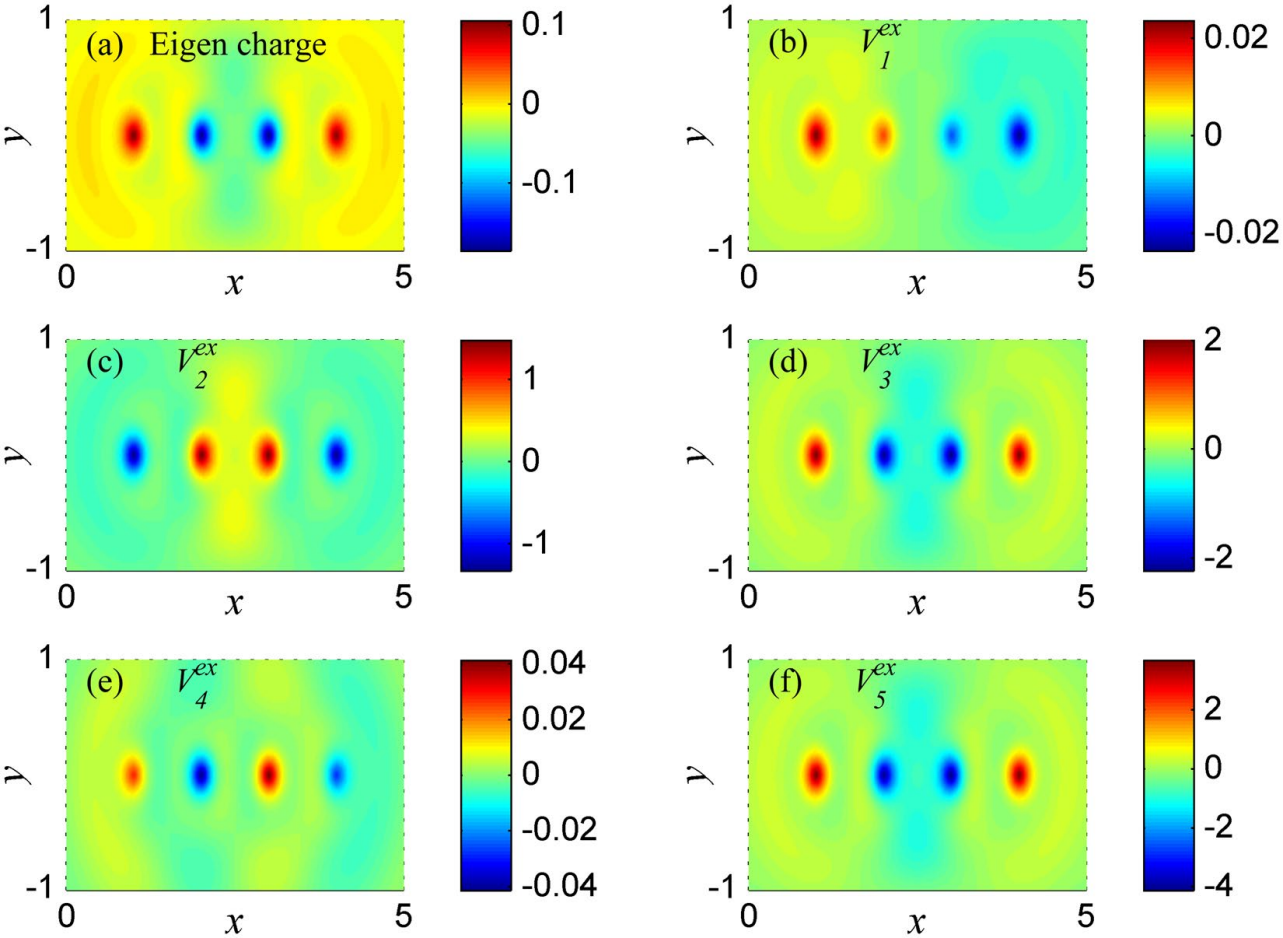
The charge distribution in the system of Fig. 2 at plasmon frequency *ω*_2_ = 2.982. (**a**) The eigen charge. (**b–f**) The charge induced respectively by external potentials ${V}_{1}^{ex}-{V}_{5}^{ex}$.

In Fig. 4(a), the eigen charge at frequency *ω*_2_ is symmetric among the atomic chain, such eigen charge has quadrupole moment along the chain direction, which implies that the plasmon at frequency *ω*_2_ is a quadrupole mode. In Fig. 4(c,d,f), the charges under potentials ${V}_{2}^{ex}$, ${V}_{3}^{ex}$ and ${V}_{5}^{ex}$ are resonated at frequency *ω*_2_, and have the same distribution characteristic as the one of eigen charge at frequency *ω*_2_. However, in Fig. 4(b,e), due to the charges under potentials ${V}_{1}^{ex}$ and ${V}_{4}^{ex}$ are not resonated at frequency *ω*_2_, the non-resonant charges are polarized by potentials ${V}_{1}^{ex}$ and ${V}_{4}^{ex}$ respectively. And the distributions of these charges are different from eigen-charge distribution at frequency *ω*_2_.

**Figure 5:**
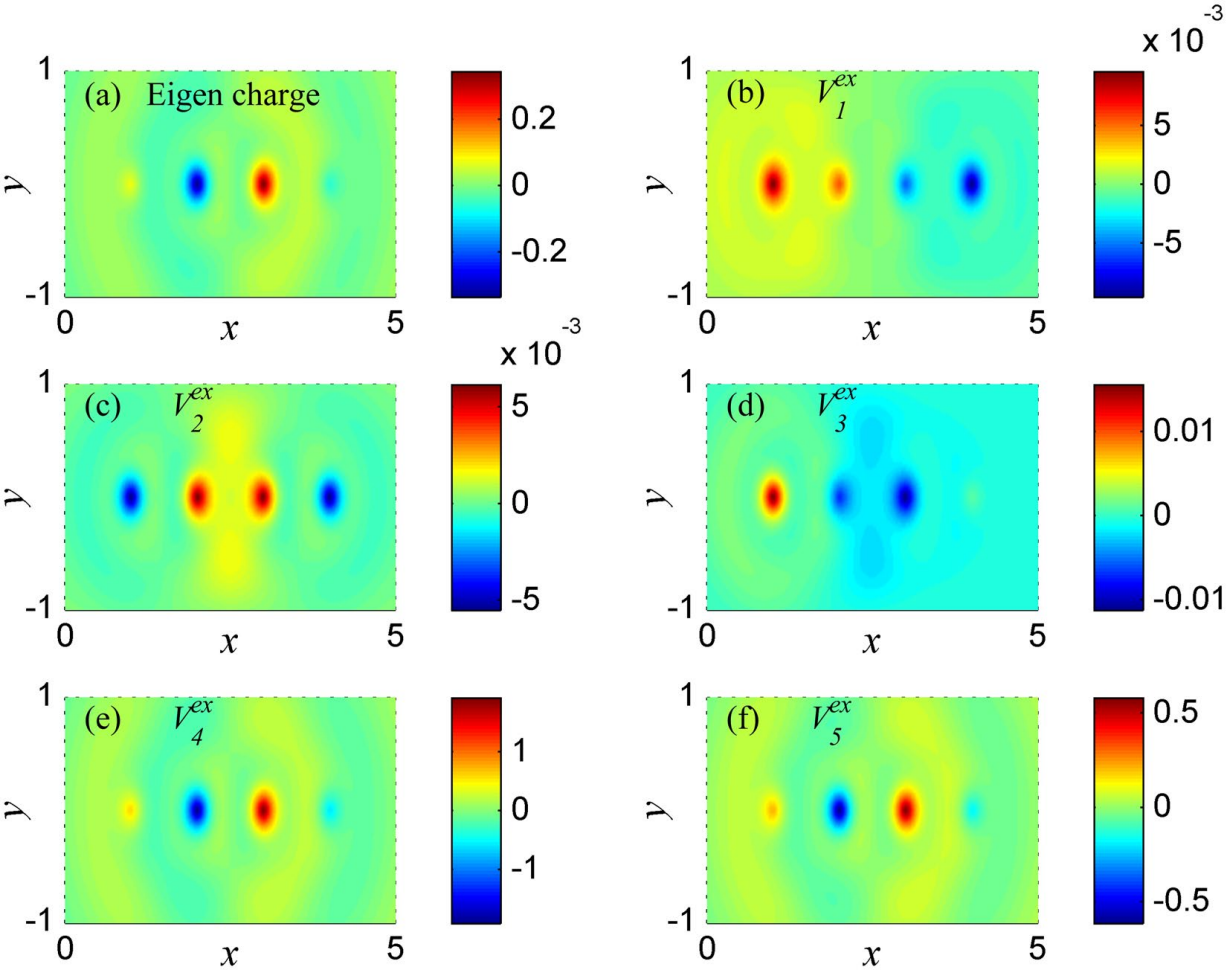
The charge distribution in the system of Fig. 2 at plasmon frequency *ω*_3_ = 4.176. (**a**) The eigen charge. (**b–f**) The charge induced respectively by external potentials ${V}_{1}^{ex}-{V}_{5}^{ex}$.

In Fig. 5(a), the eigen charge is asymmetric among the atomic chain, such distribution of charge has both dipole and quadrupole moment along the chain direction, which implies that the plasmon at frequency *ω*_3_ is a normal high order mode. In Fig. 5(e,f), the charges under potentials ${V}_{4}^{ex}$ and ${V}_{5}^{ex}$ are resonated at frequency *ω*_3_ and have the same distribution characteristic as the one of eigen charge at frequency *ω*_3_. However, in Fig. 5(b,d), the charges under potentials ${V}_{1}^{ex}$, ${V}_{2}^{ex}$ and ${V}_{3}^{ex}$ are not resonated at frequency *ω*_3_, and the distributions of these charges are different from the distribution of eigen charge at frequency *ω*_3_. The non-resonant charges under potentials ${V}_{1}^{ex}$, ${V}_{2}^{ex}$ and ${V}_{3}^{ex}$ are polarized by potentials ${V}_{1}^{ex}$, ${V}_{2}^{ex}$ and ${V}_{3}^{ex}$, respectively.

The above results showed in Figs 3–5 imply that the distribution characteristics of the resonant charge will be the same as the one of eigen charge. Here, we assume the plasmon frequency in the atomic chain is *ω*_*P*_, then when the frequency of external potential is equal to *ω*_*P*_, according to the Eq. (8), we obtain that the energy absorption $\,L({\omega }_{P})\propto \frac{1}{2}{\omega }_{P}{\rm{Im}}\{\sum _{l}{Q}^{eg}(l,{\omega }_{P}){V}^{ex}(l,{\omega }_{P})\}$, where, *Q*^*eg*^(*l*, *ω*_*P*_) is the intrinsic property of plasmon which only depends on the plasmon frequency *ω*_*P*_, and *Q*^*eg*^(*l*, *ω*_*P*_) has the same symmetry as *Q*^*eg*^(*r*, *ω*_*P*_). For a fixed plasmon mode, *Q*^*eg*^(*l*, *ω*_*P*_) is a fixed function, and the energy absorption only depends on the external potentials *V*^*ex*^(*l*, *ω*_*P*_). Thus, *L*(*ω*_*P*_) will be zero under some specific potentials *V*^*ex*^(*l*, *ω*_*P*_), and the collective oscillations will not be maintained with no energy absorption due to scattering. This is the reason why the mode of plasmon at frequency *ω*_1_ cannot be excited by ${V}_{2}^{ex}$ (see in Fig. 2), because the energy absorption *L*(*ω*_*P*_) between the antisymmetric eigen charge (see in Fig. 3(a)) and the symmetric potential ${V}_{2}^{ex}$ is zero. For the same reason, the mode of plasmon at frequency *ω*_2_ cannot be excited by ${V}_{1}^{ex}$ and $\,{V}_{4}^{ex}$, the mode of plasmon at frequency *ω*_3_ cannot be excited by ${V}_{1}^{ex}$, ${V}_{2}^{ex}$ and ${V}_{3}^{ex}$. Hence, if the eigen-charge distribution at each mode of plasmon is calculated or observed, one can control the excitation of arbitrary plasmon mode by external field.

**Figure 6:**
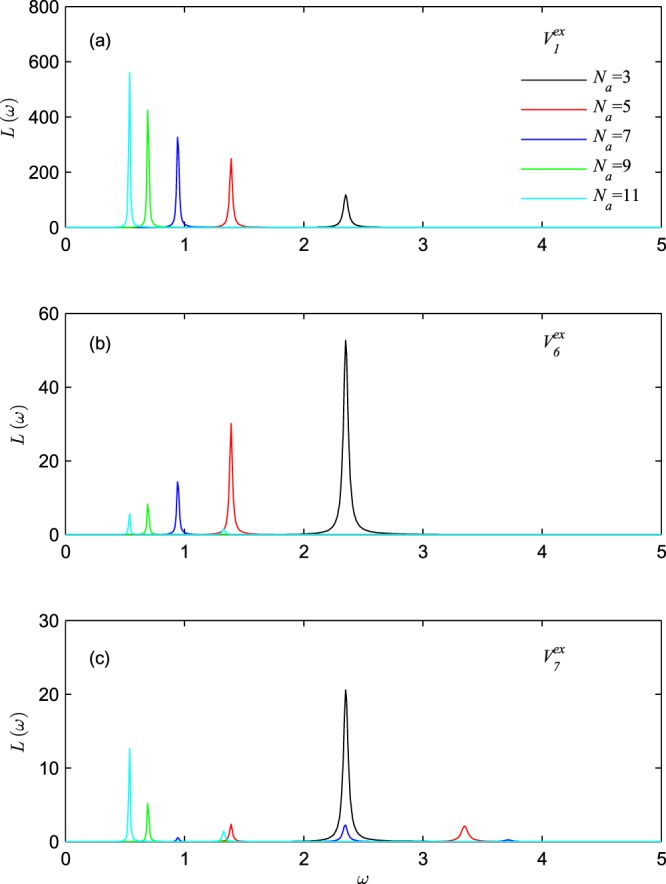
The evolution of energy absorption with atom number, where (**a**) under potential ${V}_{1}^{ex}$, (**b**) under potential ${V}_{6}^{ex}=\,-\,2(\frac{1}{l}+\frac{1}{l-{L}_{x}})$, (**c**) under potential ${V}_{7}^{ex}=\frac{1}{4}{V}_{1}^{ex}+{V}_{6}^{ex}$. The electron number is fixed to be *N*_*e*_ = 2.

Next, we further investigate the influence of external-field distribution on the evolution of energy absorption with the atom number. To be simple, Fig. 6 only show the evolution of the dipole mode of plasmon, where Fig. 6(a,b) are obtained under potentials ${V}_{1}^{ex}$ and ${V}_{6}^{ex}$, respectively. Potential function |${V}_{1}^{ex}$| is a monotone increasing function of space coordinates of atoms, potential function |${V}_{6}^{ex}$| is a monotone decreasing function of space coordinates of atoms. In Fig. 6(a,b), the plasmon frequencies shift to red with the increase of the atom number which are the same as in refs^13–16,19,20^. However, in Fig. 6(b), the intensity of energy-absorption peak (ie, plasmon-energy absorption) monotonically decreases with the increase of the atom number, where this result is abnormal and is not consistent with the normal result^13–16,19,20^. The normal result in refs^13–16,19,20^ is consistent with the result in Fig. 6(a) that the plasmon-energy absorption increases with the atom number increasing. And we notice that the normal result commonly occurs under monotone increasing potential. Then, it’s easy to find out that the difference between Fig. 6(a,b) results from the effect of the potential on the intensity of collective oscillations: under monotone decreasing potential, the collective oscillations of charge will be weaker in more-atoms system, so less energy will be absorbed to sustain the weaker collective oscillations. Moreover, the influences of external field on the evolution of energy absorption at quadrupole and other modes of plasmon are also calculated, however, these parts of results are not shown in this paper since the calculated results are similar to the one of dipole mode.

Inspired by the results of Fig. 6(b), under potential ${V}_{7}^{ex}=\frac{1}{4}{V}_{1}^{ex}+{V}_{6}^{ex}$, we have shown that the energy absorption (see Fig. 6(c)) at plasmon will not change monotonically with the atom number. So that, with the increase of atom number, there are three types of evolutions of energy absorption: the first one, the energy absorption increases monotonically; the second one, the energy absorption decreases monotonically; the third one, the energy absorption changes non-monotonically. And it’s easy to find that, the first type of evolutions occurs under monotone increasing potential, the second type of evolutions occurs under monotone decreasing potential. For quadrupole plasmon that excited by symmetric potential, we should point out that the first type of evolutions occurs as the absolute value of the external potential increases in the left half part of space. This result may provide a new idea for observing quantum mode of plasmon in tiny cluster system.

**Figure 7:**
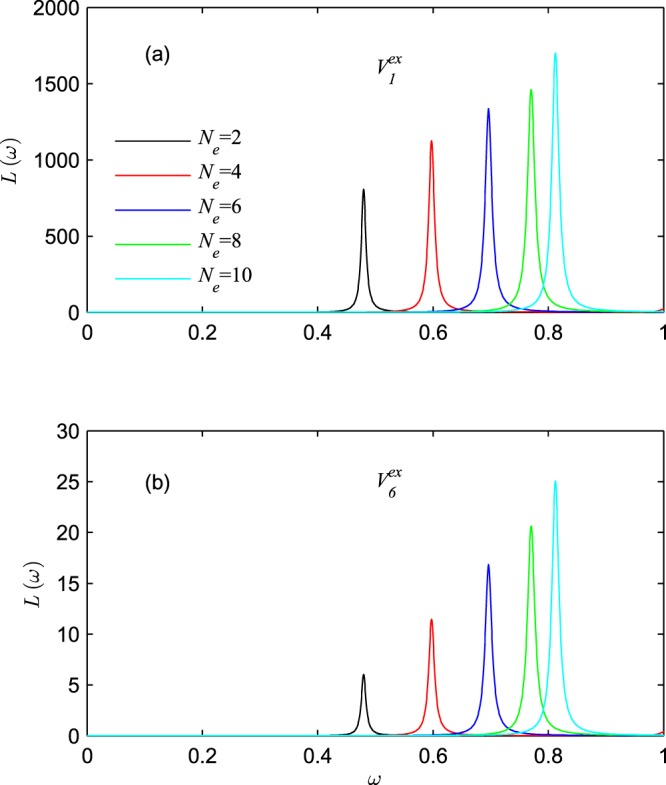
The evolution of energy absorption with electron number, where (**a**) under potential ${V}_{1}^{ex}$, (**b**) under potential ${V}_{6}^{ex}$. The atom number is fixed to be *N*_*a*_ = 10.

In Fig. 7, we further investigate the influence of external-field distribution on the evolution of energy absorption by setting different electron number and external potentials, where Fig. 7(a,b) correspond to ${V}_{1}^{ex}$ and ${V}_{6}^{ex}$, respectively. Here, besides differences in the intensity of energy absorption, the evolutions of energy absorptions are similar under potentials ${V}_{1}^{ex}$ and ${V}_{6}^{ex}$. So, regardless of any type of potential is applied to the atomic chain, the intensity of the energy absorptions at plasmon will increase with the increase of the electron number, because more electrons will participate into the collective oscillations. And result from the increase of the fermi energy, the plasmon frequencies will shift to blue with the electron number increasing.

## Conclusion

We have studied the plasmon excitation in linear atomic chains based on the tight binding model and self-consistent linear response theory. Results show that the plasmon modes are different under various types of external potentials. Each mode of plasmon corresponds to one kind of eigen-charge distribution. And, if the eigen-charge distribution at each plasmon mode is given, the excitation of arbitrary plasmon mode can be controlled by external field. Furthermore, the relationship between the plasmon-energy absorption and the atom number is influenced by external electric potential. Especially, under monotone decreasing potential, the plasmon-energy absorption may decrease with the increase of atom number. However, regardless of any type of potential is applied to the same atomic chain, the plasmon-energy absorptions will increase with the increase of the electron number. Controlling various modes of plasmon by external-field distribution has application value in new logic optoelectronic device. And the influence of external-field distribution on the evolution of energy absorption with electron number may provide a new idea for observing quantum mode of plasmon in tiny cluster system.

## References

[1] Chen W, Zhang S, Deng Q, Xu H (2018). Probing of sub-picometer vertical differential resolutions using cavity plasmons. Nat. Commun..

[2] Taylor AB, Zijlstra P (2017). Single-Molecule Plasmon Sensing: Current Status and Future Prospects. Acs Sensors.

[3] Cushing SK, Wu N (2016). Progress and Perspectives of Plasmon-Enhanced Solar Energy Conversion. J. Phy. Chem. Lett..

[4] Liu N, Tang ML, Hentschel M, Giessen H, Alivisatos AP (2011). Nat. Mater..

[5] Grasso L (2015). Molecular screening of cancer-derived exosomes by surface plasmon resonance spectroscopy. Anal. Bioanal. Chem..

[6] Andrae K, Reinhard PG, Suraud E (2004). Crossed beam pump and probe dynamics in metal clusters. Phy. Rev. Lett..

[7] Sancho-Parramon J, Bosch S (2012). Dark modes and Fano resonances in plasmonic clusters excited by cylindrical vector beams. Acs Nano..

[8] Fletcher G (2015). Multipolar and dark-mode plasmon resonances on drilled silver nano-triangles. Opt. Express.

[9] Chen F, Alemu N, Johnston RL (2011). Collective plasmon modes in a compositionally asymmetric nanoparticle dimer. AIP Adv..

[10] Zuloaga J, Prodan E, Nordlander P (2009). Quantum description of the plasmon resonances of a nanoparticle dimer. Nano Lett..

[11] Kuisma M (2015). Localized surface plasmon resonance in silver nanoparticles: Atomistic first-principles time-dependent density-functional theory calculations. Phy. Rev. B.

[12] Saito H, Yamamoto N (2015). Size dependence of bandgaps in a two-dimensional plasmonic crystal with a hexagonal lattice. Opt. Express.

[13] Yuan Z, Gao S (2008). Plasmon resonances in linear atomic chains: free-electron behavior and anisotropic screening of d electrons. Phy. Rev. B.

[14] Yan J, Yuan Z, Gao S (2007). Emergence of collective plasmon excitation in a confined one-dimensional electron gas. Phys. Rev. Lett..

[15] Cassidy A, Grigorenko I, Haas S (2008). Formation of collective excitations in quasi-one dimensional metallic nanostructures: size and density dependence. Phy. Rev. B.

[16] Muniz RA, Haas S, Levi AFJ, Grigorenko I (2009). Plasmonic excitations in tight-binding nanostructures. Phy. Rev. B.

[17] Liu DD, Zhang H (2011). A time-dependent density functional theory investigation of plasmon resonances of linear Au atomic chains. Chin. Phys. B.

[18] Liu DD, Zhang H, Cheng XL (2012). Plasmon resonances and electron transport in linear sodium atomic chains. J. App. Phys..

[19] Wu R, Xue H, Yu Y, Hu H, Liu Q (2014). Quadrupole Plasmon Excitations in Confined One-dimensional Systems. EPL.

[20] Wu R, Xue H, Yu Y, Hu H (2014). Dipole and quadrupole plasmon in confined quasi-one-dimensional electron gas systems. Phy. Lett. A.

[21] Chui ST, Bray JW (1980). Computer renormalization group calculation of the 2*k*_*F*_ and 4*k*_*F*_ correlation functions of an extended one-dimensional Hubbard model. Phys. Rev. B.

[22] Bray JW, Chui ST (1979). Computer renormalization-group calculations of 2*k*_*F*_ and 4*k*_*F*_ correlation functions of the one-dimensional Hubbard model. Phys. Rev. B.

[23] Shi T, Chang DE, Cirac JI (2015). Multiphotonscattering theory and generalized master equations. Phys. Rev. A.

[24] Asenjo-Garcia A (2017). Exponential Improvement in Photon Storage Fidelities Using Subradiance and “Selective Radiance” in Atomic Arrays. Phys. Rev. X.

[25] Toyoda T (1998). Self-consistent linear response approximation for quantum many-body systems. Physica A.

[26] Uchida T, Hiraiwa N, Yamada K, Fujita M, Toyoda T (2014). Magnetic induction dependence of the dispersion of magnetoplasmon in a two-dimensional electron gas with finite layer thickness. Int. J. Mod. Phys. B.

[27] Yu YQ, Yu YB, Xue HJ, Wang YX, Chen J (2016). Plasmon excitations in two-dimensional atomic cluster systems. Physica B.

